# Normal Value of Perfusion Index in Healthy Neonates Born in Iran

**DOI:** 10.34172/aim.31293

**Published:** 2024-11-01

**Authors:** Maryam Saeedi, Razieh Sangsari, Kayvan Mirnia, Monireh Ghanbari

**Affiliations:** ^1^Children’s Medical Center, Pediatric Center of Excellence, Tehran University of Medical Sciences, Tehran, Iran

**Keywords:** Newborn, Oxygen saturation, Perfusion index, Median PI value, PI normal range

## Abstract

**Background:**

Perfusion index is a dependable indicator for assessing the perfusion status of newborns. A low perfusion index indicates compromised hemodynamic function. The study aims to investigate perfusion index in asymptomatic newborns aged 35 to 41 weeks who did not require medical support.

**Methods:**

Healthy neonates born in four major maternity hospitals of Tehran University from 2019 to 2021 were selected. To ensure consistency and reliability in data collection, a detailed manual was developed and distributed, along with comprehensive training sessions for all personnel involved.

**Results:**

A total of 994 newborns entered the study. Among them, echocardiography was adversely affected in 218 neonates due to abnormal screening pulse oximetry. Of these 218 neonates, 53 were found to have abnormal echocardiography results. The median perfusion index value in healthy neonates was 1.6%, and the median oxygen saturation was 97%. A percentile perfusion index curve was developed for healthy neonates to establish a normal reference range.

**Conclusion:**

Developing a percentile perfusion index curve specific to healthy neonates provides a useful reference range for healthcare providers to assess perfusion status in this population, but further research is needed to confirm its accuracy.

## Introduction

 Evaluating the health of newborns becomes more effective and convenient with the use of pulse oximetry, a non-invasive tool that monitors neonatal heart rate (HR) and oxygen saturation (SpO_2_). In addition, the Perfusion Index (PI) exhibited during pulse oximetry monitoring serves as a means to assess pulse intensity by calculating the pulsatile (AC) and non-pulsatile (DC) ratio signals.^1,2^ Monitoring PI can aid in evaluating neonatal hemodynamics, particularly in critically ill infants.^3^ A decrease in PI can indicate the severity of illness, as changes in stroke volume and vasomotor tone influence this measure. Lower PI values are associated with reduced perfusion and can serve as an early detection tool for adverse outcomes during the neonatal period.^4^ Research indicates that lower PI in neonates correlates with diminished superior vena cava flow; serving as a predictor of volume status and suggesting a deterioration of the newborn’s condition.^5^ Interestingly, newborns with transient tachypnea of the newborn (TTN) may exhibit lower PI values compared to healthy counterparts. Monitoring PI offers insights into subtle changes in perfusion that may be overlooked by static displays.^6,7^ To accurately assess potential pathological conditions, it is essential to understand the normal range of PI. Studies have shown that PI can vary based on factors such as the neonate’s gender, weight,^8,9^ chronological age, and gestational age (GA).^10^ Moreover, PI can range from 0.02% to 20%, depending on the monitoring site and patient’s age.^11^ However, further research is necessary to establish the median PI in infants, particularly in healthy term neonates within the first 24 hours after birth. This study aims to map peripheral PI distribution in asymptomatic neonates with GA of 35 to 41 weeks who did not need any medical treatment. This study aims to plot PI normal range values in healthy neonates in Iran.

## Material and Methods

 This observational cross-sectional survey aimed to assess physiological indicators (PI) among asymptomatic, healthy newborns aged 35 to 41 weeks who were 24 hours old at the time of enrollment. To ensure uniformity in the studied population and increase the strength of the study, we selected four major maternity hospitals affiliated with Tehran University. Out of the six maternity hospitals associated with the university, we chose these four from different locations across Tehran. Consistency in data collection practices was maintained across all participating hospitals to enhance the reliability and validity of the research findings. To achieve this goal, we developed and distributed a detailed data collection manual that outlined standardized procedures, definitions, and methodologies for data collection. Additionally, we conducted comprehensive training sessions for all personnel involved in data collection at each hospital. Participants in the study included healthy newborns delivered either via vaginal delivery or elective cesarean section, which did not require any medical intervention at birth. The study population comprised healthy newborns delivered at the four hospitals over a two-year period, from 2019 to 2021. All newborns received standard postnatal care, facilitated by experienced midwives at each hospital, in accordance with unit guidelines. This approach aimed to eliminate confounding factors such as variability in caregiver experience and care quality, which could affect PI measurements.

 Newborns with pre- or post-ductal SpO_2_ levels less than 95%, a pre- to post-ductal SpO_2_ gradient greater than 3%, or a PI value less than 0.9 underwent echocardiography^12^ and were excluded from the study. Additionally, neonates presenting with congenital anomalies or those who were discharged before reaching 24 hours of life were also excluded from the study. Healthy neonates were defined as those not requiring resuscitation or medical support after birth, those not born with meconium-stained amniotic fluid, and those who did not die within two days of delivery.

 The pediatric resident utilized a Masimo Pulse Oximeter (Radical-7, USA) to document PI, HR, and SpO_2_. The palm or wrist on the right side was the chosen placement for pre-ductal monitoring, while either foot was used for post-ductal monitoring. SpO_2_ and HR values were recorded when the pulse oximeter displayed stable waveforms, along with the accompanying PI values. In cases where PI values were invalid ( ≤ 0.02 or ≥ 20%), measurements were repeated until valid values were obtained. Additionally, demographic data such as sex, birth weight (BW), and GA were recorded for each participant. Sample size calculation followed the methodology of Hu and colleagues’ study,^10^ which reported a standard deviation (SD) of 1.26. With a margin of error (d) set at 0.08, a total sample size of 953 patients was determined for adequate statistical power.

###  Statistical Analysis

 We used SPSS 26.0 for Windows for analysis. The paired *t* test was used to compare characteristic data. Multiple linear regressions showed PI value relationship with SpO_2_, while adjusting for sex, birth weight, and GA. Statistical significance was determined as *P* < 0.05. To illustrate the variation of PI between GAs in a more meaningful way, percentile curves were created. Cole’s Lambda Mu Sigma (LMS) method was applied to plot these curves at the 3rd, 10th, 25th, 50th, 75th, 90th, and 97th percentiles. The LMS method utilizes the Box-Cox transformation of the three parameters to achieve a near-normal distribution of the PI measurement. The L parameter introduces a non-linear change to PI, the M parameter represents the mean of the normal distribution, and the S parameter represents the coefficient of variation. As the covariate shifts, the three parameters must adjust in a consistent manner.^13^ LMS chart maker is a program to fit smooth centile curves to reference data using the LMS method.^10^ Non-linear regression combined with penalized likelihood allows the three curves to be modeled as cubic splines, and the amount of smoothing required can be represented by smoothing parameters or the same number of freedoms.^14^ The process consists of five stages, namely data entry, model fitting, graphical display, model checking, and model saving, with the last four stages being repeated as many times as required.

 The software used for this analysis and modeling was the LMS chart maker program developed by Pan H and Cole TJ. version 2.54. The program was obtained from the website https://lmschartmaker.software.informer.com/2.5/ in 2011.

## Results

**Figure 1:**
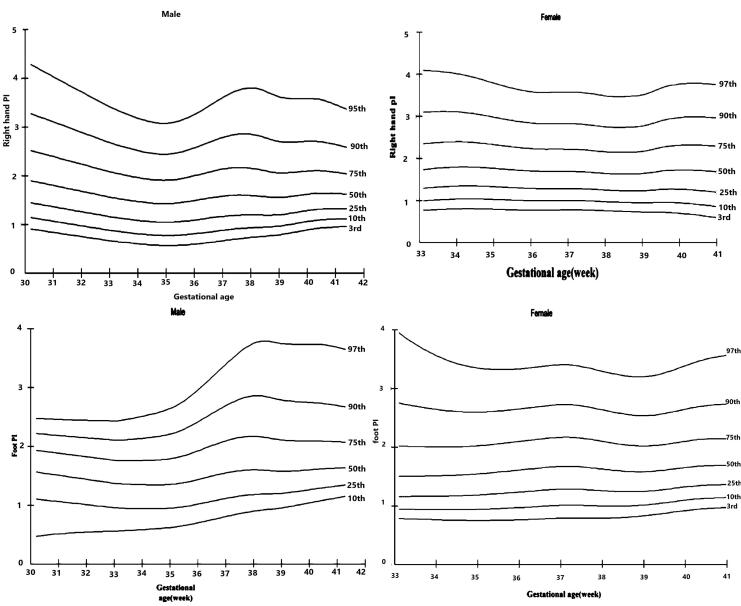
Percentile Pre- and Post-ductal PI Curve in Healthy Male and Female Neonates

 In our study, 994 newborns with a GA of 35‒41 weeks entered the study. Of these newborns, 49.9% were female and 50.1% were male. The average GA was 38.5 ± 1.26 weeks. The mean BW was 3209 ± 449 grams. Among the newborns, 218 had abnormal pulse oximetry screening, pre- or post-ductal SpO_2_ < 95%, the pre-post ductal SpO_2_ gradient > 3%, or the PI < 0.9 and an echocardiography was performed. In these cases, 53 newborns had abnormal echocardiography findings. In healthy newborns, both pre- and post-ductal PI had a median value of 1.6%. The median values of both pre- and post-ductal arterial oxygen saturation (SPO_2_) in healthy newborns were 97%. In neonates with congenital heart defect (CHD), the median pre-ductal and post-ductal PI was 1%, and the median pre-ductal and post-ductal SPO_2_ was 95% (Table 1). The mean pre-ductal and post-ductal PI was calculated based on the presence of cardiac disease. The mean of PI showed a significant relationship with the presence of CHD (pre-duct *P* value = 0.001) and (post-duct *P* value = 0.001), but there was no significant relationship with other variables (Table 2). Pearson’s correlation coefficients were used to determine the relationship between PI and SPO_2_. This analysis revealed a strong positive relationship between pre-ductal SPO_2_ and pre-ductal PI in neonates with CHD (r = 1, *P* = 0.04) and a strong positive relationship between post-ductal SPO_2_ and PI in healthy neonates (r = 1, *P* = 0.03). In both cases, higher SPO_2_ was associated with higher PI (Table 3). The mean pre-ductal and post-ductal PI in healthy neonates were 1.8% and 1.7%, while the mean pre-ductal and post-ductal PI in neonates with CHD were 1.17% and 1.3%, respectively (Table 1). Given the sample size and the obtained information, we created a percentile PI curve in healthy neonates to be used as a normal reference value (Figure 1).

**Table 1:** Distribution of Pre- and Post-ductal PI and SPO_2_

		**Right Hand Sat**	**Foot Sat**	**Right Hand PI**	**Foot PI**
Healthy	N	941	941	941	941
	Mean	97.014	97.0478	1.8340	1.7751
	Median	97.000	97.0000	1.6000	1.6000
	Standard deviation	2.0172	2.19788	0.81782	0.74569
	Minimum	88.0	63.00	0.09	0.30
	Maximum	100.0	100.00	7.90	7.50
CHD	N	53	53	53	53
	Mean	95.642	95.7925	1.1732	1.3019
	Median	95.000	95.0000	1.0000	1.0000
	Standard deviation	3.3345	3.62342	0.68143	0.81700
	Minimum	82.0	78.00	0.08	0.10
	Maximum	100.0	100.00	4.50	5.20

CHD, Congenital heart defect; PI, Perfusion index; SPO_2_, Oxygen saturation.

**Table 2:** Linear Regression for Pre- and Post-ductal PI

**Model**	**Unstandardized Coefficients**		**Standardized Coefficients**	**T**	**Significant**	**95% CI**
	**B**	**Standard Error**	**Beta**			
**Dependent Variable: Right hand PI**						
Echocardiography	-0.630	0.088	-0.226	-7.141	0.0000	-0.803, -0.457
Gender	0.0530	0.040	0.042	1.308	0.1910	-0.26, 0.132
GA (wk)	0.0150	0.018	0.028	0.799	0.4250	-0.21,0.051
BW (kg)	0.0570	0.049	0.040	1.158	0.2470	-0.39, 0.153
**Dependent Variable: Foot PI**						
Echocardiography	-0.459	0.0820	-0.179	-5.614	0.0000	-0.62, -0.299
Gender	0.022	0.0370	0.019	0.5990	0.5490	-0.51, 0.096
GA (wk)	0.018	0.0170	0.037	1.056	0.2910	-0.015, 0.052
BW (kg)	0.047	0.0450	0.036	1.029	0.3040	-0.042, 0.136

B, beta coefficient; T, T-value; PI, Perfusion index.

**Table 3:** Correlation between SPO_2_ and PI

			**Right Hand Sat**	**Right Hand PI**
Healthy	Right hand sat	Pearson Correlation	1	-0.060
		Sig. (2-tailed)		0.064
	Right hand PI	Pearson Correlation	-0.060	1
		Sig. (2-tailed)	0.064	
CHD	Right hand sat	Pearson Correlation	1	-0.282*
		Sig. (2-tailed)		0.040
	Right hand PI	Pearson Correlation	-0.282*	1
		Sig. (2-tailed)	0.040	
Healthy	Foot sat	Pearson Correlation	1	-0.069*
		Sig. (2-tailed)		0.034
	Foot PI	Pearson Correlation	-0.069*	1
		Sig. (2-tailed)	0.034	
CHD	Foo. sat	Pearson Correlation	1	-0.169
		Sig. (2-tailed)		0.227
	Foot PI	Pearson Correlation	-0.169	1
		Sig. (2-tailed)	0.227	

* Correlation is significant at the 0.05 level (2-tailed). CHD, Congenital heart defect, PI, Perfusion index.

## Discussion

 In healthy neonates, skin perfusion is higher than the oxygen demand. However, when illness occurs, the body reallocates cardiac output to ensure that essential organs such as the brain, heart, and adrenal glands receive sufficient oxygen. This period, before perfusion reduction becomes irreversible, is known as the golden time. Identifying neonates during this stage can enable prompt treatment initiation.^13,14^ Pulse oximetry PI is being increasingly recognized as a useful parameter to enhance sensitivity in detecting significant neonatal complications. Many studies concentrate on utilizing PI to forecast life-threatening illnesses.^4,15^ Given the potential of PI in diagnosing the severity of a newborn’s disease and screening for heart conditions, it is essential to study the normal range of PI in healthy neonates.

 In 2017, Jegatheesan et al conducted a study in the United States that employed a computerized data selection method to find the median PI in asymptomatic neonates who were 24 hours old. Their study yielded a median PI of 1.8%, displaying a tight interquartile range (IQR) between 1.2 and 2.7.^16^ The nomogram in Granelli and Ostman-Smith study’s, which reported data from asymptomatic newborns at 1 to 120 hours of life, showed a median PI of 1.7% and an IQR of 1.18 to 2.5. These findings are consistent with our own research that showed PI with a median of 1.6% and an IQR of 1.3 to 2.1. These consistent distributions of PI suggest that PI values in asymptomatic neonates exhibit a limited range of distribution during screening time.^17^ Hawkes and colleagues found that PI had a wider range during the first 5 minutes after birth compared to the period after.^18^

 Our study showed that the median of pre-ductal and post-ductal PI in healthy neonates was 1.6%, and the median of pre-ductal and post-ductal SPO_2_ in healthy neonates was 97%. Although the Jegatheesan et al study reported a post-ductal PI that was 0.1 lower than the pre-ductal PI,^16^ and the Granelli and Ostman-Smith’s study showed a post-ductal PI that was 0.02 higher than the pre-ductal PI^17^, Hua et al reported a median pre-ductal and post-ductal PI of 1.7%.^19^ These small differences depend on the chronological age and the GA of the studied healthy newborns.

 In our neonates with CHD, the median of pre- and post-ductal PI was 1%. The mean of PI had a significant relationship with the presence of heart disease (pre-ductal *P* value = 0.000) and (post-ductal *P* value = 0.000). A study conducted by Iuri Corsini and colleagues reported a significant correlation between the PI and left ventricular output in term infants.^20^ Schena et alconducted a prospective study in 16 hospitals where a total of 42,169 asymptomatic newborns were screened using pre- and post-ductal SPO_2_ and PI. The study concluded that PI can be useful in identifying cases of coarctation of the aorta that may have been missed by pre- and post-ductal SPO_2_ measurements.^12^ In another cross-sectional study in patent ductus arteriosus neonates, a significant difference was observed in pre- and post-ductal PI before and after arterial duct closure (*P* = 0.004).^21^ Most of the studies are related to the PI and left heart disorders. The emphasis is on the diagnosis of left heart diseases that cannot be accurately diagnosed solely by pre- and post-ductal SPO_2_ measurements,^22^ but in our study there was no preference for left heart disease. A case of total anomalous pulmonary venous return had pre-ductal PI of 1% and post-ductal PI of 0.9%. Although these PIs are higher than the number required for the screening of CHD, they are actually lower than the normal range typically found in healthy newborns. Therefore, this study also emphasizes the inclusion of PI in pre- and post-ductal SPO_2_ measurements for CHD screening in healthy neonates.

 In this study, we designed percentile PI curves in healthy neonates to be used as normal values (Figure 1). In 2020, a study carried out in China provided data on the percentiles of pre- and post-duct PI for neonates with GA of 35 to 41 weeks, with a specific focus on sex differences.^8^ The presented percentile curves can aid medical professionals in determining the standard PI value depending on the GA and gender. This can potentially lead to the early identification of high-risk neonates.

## Limitation

 This study faced some limitations that, if addressed, could have led to more thorough results. Notable limitations included possible biases in data collection, the individuals responsible for data collection were not identical, and variations in demographics. Initially, an extended follow-up period for the newborns might have offered valuable information regarding their health, making it a crucial aspect of the study. Additionally, a larger sample size could have been included, which would have increased the statistical power and reliability of the findings.

## Conclusion

 The newborn-specific PI percentile curve is important for neonatologists as it aids in the early diagnosis of high-risk neonates. However, further research is necessary to validate the accuracy of this curve.
